# Antiretroviral Therapy Interruption (ATI) in HIV-1 Infected Patients Participating in Therapeutic Vaccine Trials: Surrogate Markers of Virological Response

**DOI:** 10.3390/vaccines8030442

**Published:** 2020-08-05

**Authors:** Lorna Leal, Csaba Fehér, Valèria Richart, Berta Torres, Felipe García

**Affiliations:** 1 Infectious Diseases Department—HIV Unit, Hospital Clínic Barcelona, IDIBAPS, University of Barcelona, 08036 Barcelona, Spain; feher.csaba@gmail.com (C.F.); valeria.richart.sierra@gmail.com (V.R.); btorres@clinic.cat (B.T.); fgarcia@clinic.cat (F.G.); 2 AIDS Research Group, IDIBAPS, Hospital Clinic, University of Barcelona, 08036 Barcelona, Spain

**Keywords:** antiretroviral therapy interruption, ATI, HIV-1, surrogate markers, response

## Abstract

A functional Human immunodeficiency Virus (HIV) cure has been proposed as an alternative to antiretroviral treatment for life, and therapeutic vaccines represent one of the most promising approaches. The goal of therapeutic vaccination is to augment virus-specific immune responses that have an impact on HIV viral load dynamics. To date, the agreed feature to evaluate the effects of these therapeutic interventions is analytical antiretroviral treatment interruption (ATI), at least until we find a reliable biomarker that can predict viral control. Different host, immunologic, and virologic markers have been proposed as predictors of viral control during ATI after therapeutic interventions. This review describes the relevance of ATI and the different surrogate markers of virological control assessed in HIV therapeutic vaccine clinical trials.

## 1. Introduction

Antiretroviral therapy (ART) is effective in maintaining viral suppression and reducing morbidity and mortality in patients with Human Immunodeficiency Virus (HIV) infection. Nevertheless, some patients have adherence problems with long-term ART, especially due to its adverse effects, leading to an increased risk of developing drug resistances. In addition, ART is a life-long therapy because the virus persists indefinitely in latent reservoirs, and there are currently no effective strategies to eliminate them [1]. There are only two individuals in the world who have ever been cured of HIV. The first individual, known as the “Berlin patient”, had acute myeloid leukemia and HIV-1 infection, and consequently received an allogeneic hematopoietic stem cell transplant from a donor with a homozygous mutation in the HIV coreceptor CCR5 (CCR5 ∆ 32 deletion). After [2] the transplant, HIV-1 remission was maintained [3]. A second individual receiving a hematopoietic stem cell transplant with the same mutation has recently been cured as his HIV viral load remains 30 months after ART interruption [4]. Nevertheless, this treatment involves a high risk of mortality and is only used in patients who also have life-threatening hematological malignancies. HIV-therapeutic research nowadays focuses on achieving a functional cure. A functional cure is the generation of an efficient host immunity capable of suppressing HIV replication in the absence of ART. This field currently lacks non-viral biomarkers of reservoir reduction or virologic control, thus requiring analytical antiretroviral treatment interruptions (ATI) to assess in-vivo efficacy of interventions aiming to achieve ART-free HIV remission [5]. The safety of this strategy has extensively been analyzed [5]. Due to the huge diversity of novel interventions in preclinical or early-phase clinical studies requiring efficacy assessment, ATI are expected to rise in importance. However, the current SARS-CoV2 pandemic has changed the playing field and the feasibility and safety of performing ATI in the coming months or years is not clear. Experts advise maintaining ART even if patients are participating in clinical trials and recommend finding other ways or biomarkers to assess efficacy instead of ATI. A good surrogate marker of viral load response after ATI is now of paramount relevance. We will review the current state-of-the-art regarding ATI and how host, virological, immunological, and other factors can influence viral load (VL) rebound. 

## 2. Utility of Analytical Antiretroviral Treatment Interruption (ATI)

When ART combinations first came into use, there was uncertainty regarding how to balance adverse effects with the benefits of such treatment. This uncertainty created disagreement among experts and thus guidelines were constantly changing as to when HIV-infected individuals should start ART and whether ART should be taken continuously or intermittently. To address this issue, trials involving structured treatment interruptions (STI) were performed to outweigh the benefits and risks. STI were generally long interruptions and reintroducing ART was set to low levels of CD4+ T cells (mainly <250 cells/μL). It was first hypothesized that treatment interruptions could decrease ART toxicity and potentiate immunological response to HIV. However, these trials demonstrated a considerable increase in morbidity, mortality, and disease progression, and an increased risk of HIV transmission. Therefore, the initial hypothesis was rejected, and STI were no longer standard of care [6,7]. The only main effective treatment nowadays is ART. Nevertheless, ART does not provide a functional cure, thus new interventions aiming at virological control without ART are being evaluated. There are currently no adequate surrogate markers of treatment efficacy [8], therefore, direct assessment of viral control during ATI remains the most important clinical endpoint [9,10]. In order to mitigate risks and increase safety, ATI duration is set to virological endpoints, not clinical events, and patient control is very strict. Nonetheless, ATI are only justifiable if the participant understand the risks, and if there is a scientific question that cannot be solved efficiently by any other means [5].

## 3. Types of ATI: Virological Outcome Measures

There are two main strategies that can be used for ATI. The first one is extended interruption, which is a fixed period of treatment interruption, normally between 12 or 24 weeks, with the goal of measuring how effectively VL is controlled once it declines to a steady state, referred to as the set point. Viral set point is one of the most appropriate virological outcome measures to assess immunological interventions aiming to improve viral control after ART is interrupted. Results of successful cure and remission experiments in SIV/macaque models demonstrate that certain interventions induce VL containment for extended periods, but only after a significant increase in SIV replication after ART withdrawal [11]. This same effect has been observed in elite controllers and post-treatment controllers [12,13]. Therefore, it is assumed that virus control will be needed for any intervention aiming to induce viral suppression in the absence of ART, and in order to assess this, viral set point can be used as the primary endpoint [8]. The second strategy is a type of ATI often known as monitored antiretroviral pause (MAP), where ART is restarted at the moment of viral rebound. This virological endpoint is known as time to viral rebound (TtR) and is considered the safest endpoint in ATI protocols. It involves frequent monitoring of plasma HIV RNA with real-time measurements until VL rebound is confirmed. Its main advantage is that by reducing its duration, it may prevent potential complications. The conceivable downside of this approach is that it will reduce the capacity to test the effectiveness of interventions that aim to work through immunological mechanisms and, consequently, might prevent the identification of virological controllers [8,10,14]. MAP are thus useful in assessing strategies aiming to reduce reservoir size [15].

There is no “gold standard”, so studies tend to use TtR or viral set point depending on the study’s objective. Consequently, the comparison of efficacy in interventions between ATI studies is highly difficult. Nevertheless, there are few available publications that directly address the correlations between different virological endpoints measured during ATI [8].

In one study, Treasure G et al. assessed the relationship among VL outcomes in HIV ATI trials. A total of 235 participants undergoing ATI in six AIDS Clinical Trials Group (ACTG) studies were analyzed. There was no significant association between TtR and ATI VL set point in participants who had viral rebound at or before week 12. However, patients who rebounded after week 12 had significantly lower ATI VL set point, suggesting the presence of an immunologic and/or virologic factor that could have an impact on VL dynamics. Participants who were treated during early infection were also found to have a lower ATI VL set point; a finding consistent with other studies that favor treatment from early stages of infection. Moreover, VL set point tended to be lower than pre-ART VL [14].

**Figure 1:**
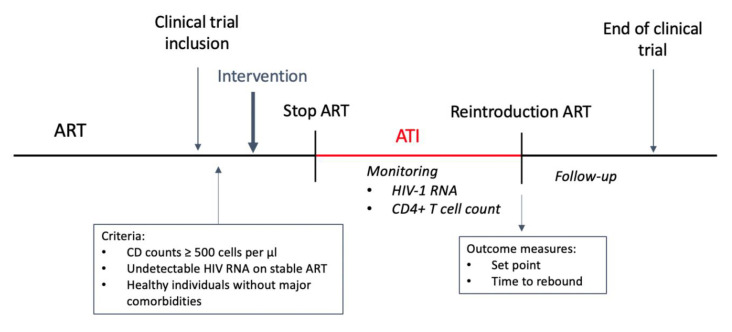
ATI scheme. Intervention can include multiple doses throughout the trial. Immunological response is assessed during ATI.

Feher et al. [8] conducted a retrospective analysis of VL evolution in 334 ATI episodes in HIV-1 chronic infected patients collected from 11 prospective studies, in order to establish correlations between different rebound parameters. The most frequently used virological outcome measures were correlated and analyzed. Their results showed that the set point during ATI was lower than the baseline VL in >60% ATI. They concluded that TtR correlated with all other quantitative and temporary outcomes and thus could possibly estimate the expected values of these later parameters. They demonstrated that clinically significant virus control is to be expected mainly in patients with longer TtR and proposed that patients with early viral rebound should be put back on ART while participants with longer TtR could be exposed to prolonged ATI. This observation opens up the possibility of TtR as an optimal marker of response in HIV cure strategies (Figure 1).

## 4. Potential Adverse Effects

The viral reservoir not only makes research on HIV remission or cure challenging, but also affects the ability to estimate risks from ATI themselves. This is due to the lack of evidence as to the size and composition of individual viral reservoirs, and thus the potential impact of ATI cannot be well evaluated. The absence of risk assessment is one of the main concerns of ATI, which are mainly based on theoretical and experienced risks [9] (see Table 1).

### 4.1. Clinical Risks

Clinically significant adverse events have been reported in treatment interruption studies, probably related to the increase of VL. The described adverse events (AEs) found in the bibliography occurred during the ATI phase or during short-term follow-up. It is important to note that many of these studies used STI and thus interruptions had a longer duration, endpoints were more flexible, and inclusion criteria were less strict. Reported clinical adverse events include acute retroviral syndrome, cardiovascular, renal, or hepatic disease, among others.

Symptoms resembling those of acute retroviral syndrome may occur during ATI due to viral rebound. Patients more frequently presented fever, lymphadenopathy, rash, myalgia, diarrhea, headache and pharyngitis. However, symptoms resolved quickly upon resuming treatment [16,17,18,19]. Thrombocytopenia was also described in several studies [18,20,21].

The SMART group randomized over 5000 participants to continuous or interrupted ART to investigate whether ART could be used intermittently and maintain CD4+ T cell counts above a threshold. Participants in the interrupted ART arm were to reinitiate ART when CD4+ count decreased to <250 cells/mm^3^ until an increase to over 350 was achieved. The results demonstrated that although the overall rate of events was relatively low, the risk of serious illness and mortality was more than double among those receiving intermittent ART. Higher incidence of opportunistic diseases and death from other causes were found in the interrupted ART groups and thus confirmed that immunosuppression increased the risk of death. They also showed that participants undergoing a STI had a higher rate of major cardiovascular, renal, or hepatic disease than those in the viral suppression group. Part of the difference in the rates of opportunistic diseases or death from any cause between participants in STI and the viral suppression group was explained by the differences in the CD4+ T cell count and HIV RNA level [6]. The large size of this study and the strength of its evidence had a major impact on treatment guidelines as they recommended continuous ART and early initiation of treatment for HIV-infected individuals. Subsequent analyses of the SMART study revealed that biological markers related to inflammation (IL-6 and D-dimer) were associated with the risk of adverse effects. This demonstrated that rising levels of VL during STI increased inflammation levels, thus becoming a key contributing factor to serious complications [22].

Neurological events are poorly defined risks of ATI. However, previous experience with cerebrospinal fluid monitoring during prolonged ART interruption indicated that rebound HIV RNA accompanied by elevations in biomarkers of intrathecal inflammation and neuronal injury were found approximately 20 days after ATI [23].

Many of the short-term clinical adverse events have been thoroughly studied in the past years. Due to this research, the use of STI are nowadays restricted to ATI in clinical trials to evaluate efficacy. Data from these large studies involve extensive treatment interruptions or delayed ART, and therefore, are difficult to extrapolate to time-limited, closely monitored ATI in current clinical trials. The main concerns nowadays are two potential effects of ATI, diminished responsiveness to future cure interventions, and increased chronic inflammatory processes, due to expanded viral reservoirs, even after reintroduction of effective ART [9]. In a systematic review and meta-analysis, Stecher et al. evaluated the safety of both STI and ATI. The study design, duration, and criteria for restarting ART and follow-up after interruptions differed among the 22 studies evaluated; however, they concluded that studies with narrow follow-up intervals (≤14 days) did not show a substantial increase of adverse events during treatment interruptions. Viral resistance and disease progression were the most common AEs observed. These findings indicate that ATI may be a safe strategy as part of HIV-1 cure trials by closely monitoring for viral rebound [7]. In addition, a systematic review of 159 clinical studies published from 2000–2017 performed by Lau et al. [10] concluded that ATI in recent intervention studies conducted since 2014 were more closely monitored, had more conservative thresholds to restart ART, and had a shorter treatment interruption duration compared with older studies. Both metanalyses found that the incidence of treatment interruption related AEs was low and did not cause irreparable harm to study participants. The identified factors associated with less chance of AEs were baseline CD4+ T cell >500, a shorter period of time off ART (<14 days, lowest risk), closer participant follow-up during interruption (every 14 days, lowest risk), and more conservative thresholds to restart ART [24].

### 4.2. Effects in Virologic and Immunologic Parameters

The latent HIV-1 reservoir is one of the primary targets for virus eradication and curative strategies. Upon discontinuation of ART, safety concerns related to the expansion of latent reservoir emerge. Therefore, it has been widely studied in these past years. Evidence to this date has concluded that short-term ATI does not lead to expansion of the persistent HIV reservoir [5]. Clarridge [25] et al. investigated the effect of rebounding virus following ATI on the dynamics of HIV reservoirs using longitudinal specimens collected from 10 HIV-infected individuals, who previously participated in a passive antibody transfer study. They showed that HIV reservoir size and immune parameters returned to pre-ATI levels 6–12 months after the study participants resumed ART. In addition, there was no evidence drug resistance mutations within intact HIV proviral DNA sequences following ART resumption. Salantes et al. [26] conducted a study evaluating the impact of transient viremia on the latent reservoir in participants who underwent ATI and at least six months of subsequent viral suppression in a clinical trial. The results demonstrated that transient viremia during ATI does not substantially change the size or diversity of the latent peripheral reservoir. In addition, Strongin et al. [27] validated a DNA size-exclusion (HIDE) assay for measuring the levels of integrated HIV DNA in participants of the AIDS Clinical Trials Group. For the majority of individuals, integrated DNA levels increased during ATI and subsequently declined six months after ART resumption. They concluded that there was no significant difference in the levels of integrated HIV DNA between the pre- and post-ATI time points, and thus short-term ATI can be conducted without significant impact on levels of integrated proviral DNA. Adding to these results, Papasavvas et al. [28] used peripheral blood mononuclear cells (PBMC) from 23 ART-suppressed, chronically HIV-1-infected subjects, and examined them before and during ATI and after ART resumption. Upon ART resumption, plasma VL suppression occurred after a median of 13 weeks and resulted in restoration of pre-ATI CD4+ T cells, T cell activation, and levels of cell-associated DNA and RNA. They concluded that viremia, immunologic status, and immune activation during ATI did not change persistent cellular HIV-1 RNA or total HIV-1 DNA once ART is resumed and VL suppressed. Finally, Montserrat et al. [29] assessed viral reservoir after long-term ATI (48 weeks) in chronic HIV-1 infected patients and showed that total HIV-1 DNA concentrations increased during ATI and then returned to pre-ATI levels after 104 weeks of ART resumption. However, integrated HIV-1 DNA remained elevated after reinitiating ART and at least for the duration of the study follow-up (104 weeks). Although these results could warn of a potential persistent elevation in levels of integrated HIV DNA after ART resumption, it is important to note these treatment interruptions were unusually long. All this evidence creates a strong consensus demonstrating little, if any, irreversible, virologic, immunologic, or immune activation harm resulting from ATI. Therefore, it is safe to say that ATI does not cause expansion of persistent HIV reservoir nor irreversible damage to the immune system [24].

### 4.3. Drug Resistance

One of the main adverse effects analyzed in studies is drug resistances. Previous studies with STI reported new cases of drug resistances. However, it has been revealed that the incidence of resistance mutations was higher when CD4+ T cell count was <500 cells/μL [7]. Nonetheless, another study indicated that the development of HIV drug resistances was not associated with advanced HIV or immunological decline, but is a recognized consequence of treatment interruption, especially when the interrupting regimen contained non-nucleoside analog reverse-transcriptase inhibitors (NNRTI). This is due to the fact that these drugs have long half-lives, which results in delayed wash-out and can predispose to developing drug resistances during interruption [10,30]. Consequently, prior to ATI, participants on such regimens should be switched to short-acting antiretrovirals (e.g., integrase inhibitors) 2–4 weeks before treatment interruption [5,9]. One recent study reported that ATI was not associated with an accumulation of intact proviral sequences that encode for antiretroviral drug resistance mutations that could potentially compromise treatment responses upon reinstitution of ART [25]. Another study supports this by concluding that ATI caused little drug resistance [16].

### 4.4. Epidemiological Risks: HIV Transmission

An increased risk of HIV transmission to sexual partners during treatment interruption can occur due to increased VL. Evidence of detection of HIV in semen has been documented [24]. Moreover, there have been at least two reported cases of HIV transmission to a sexual partner during ATI; one case in a therapeutic HIV vaccine trial known as the (LIGHT) study [31] and a second case in a therapeutic vaccine clinical trial in Barcelona [32]. To minimize the risk of transmission to sexual partners, participants should be clearly and comprehensively counselled on transmission risks, the use of appropriate barrier protection, and/or use of pre-exposure prophylaxis (PrEP) [5,9].

## 5. Study Design

ATI mostly depend on the trial study, and therefore one single guideline for its design is not feasible. In order to expose patients to ATI, predefined goals must be achieved for the intervention being analyzed. This means that pre-existing data should support the intervention, and thus ATI should not be merely used to generate hypotheses. In a review by Julg et al. [5], a meeting of experts from different groups discussed the main challenges concerning ATI studies and formulated recommendations on risk mitigation and monitoring.

The major inclusion criteria included stable CD4+ counts ≥500 cells/mm^3^, fully suppressed HIV RNA while on stable ART, and otherwise healthy individuals without major comorbidities.

Exclusion criteria recommendations were:

(a) Active or chronic hepatitis B virus infection and active hepatitis C infection;
(b) Active Mycobacterium tuberculosis infection;
(c) History of cancer (often with exceptions for basal cell or squamous cell carcinoma of the skin or low grade anal or cervical dysplasia);
(d) History of HIV-associated dementia or progressive multifocal leukoencephalopathy;
(e) Resistance to two or more classes of antiretroviral drugs;
(f) History of cardiovascular event or at high risk of an event;
(g) History of AIDS-defining illness/CDC category C events;
(h) History of CD4+ nadir <200 cells/mm^3^ during chronic stages of infection;
(i) Women who are pregnant or breastfeeding;
(j) Advanced non-alcoholic fatty liver and advanced non-alcoholic steatohepatitis if evidence for substantial fibrosis or evidence of cirrhosis; and
(k) HIV-related kidney disease or moderate-to-severe decrease in estimated glomerular filtration rate (<45–60 mL/min/1.73 m^2^).

The consensus reached by Julg et al. as to ATI monitoring includes the following: HIV RNA monitoring weekly for 12 weeks and then every other week, CD4+ T cell count monitoring every two weeks, clinical symptoms monitoring, and monitoring the participants’ psychosocial experiences [5]. With the use of these recommendations in clinical trials, ATI risks should be minimal, and its benefits may surpass individual risk.

## 6. Surrogate Markers of Viral Response during ATI

### 6.1. Host, Viral Load, and CD4+ T Cells Counts Influence Viral Load Rebound

Some of the characteristics of the host or the clinical stage of HIV-1 infection can influence VL rebound. There are several studies that demonstrate that both pre-ART VL and CD4+ T cell count influence the response to immunotherapy in chronic HIV-1 infected patients (Table 2). Autran et al. [33] found, in a randomized, double-blind, placebo-controlled, phase II study of vCP1452 immunization, that patients with a lower nadir CD4+ T cell count had a shorter time to ART resumption. These data have been confirmed by Huang et al. [34] in a randomized, double-blind, placebo-controlled phase 2 clinical trial of Vacc-4x, a peptide-based therapeutic HIV-1 p24Gag vaccine candidate, where CD4+ T cell count was correlated with study end-point after ATI. In these two studies [33,34] and in a randomized, placebo-controlled trial involving HIV-1-infected participants who received a recombinant adenovirus serotype 5 (rAd5) HIV-1 gag vaccine or placebo [35], those patients with higher pre-ART viral load showed a bad outcome with shorter time of VL rebound. On the other hand, no evidence of effect of CD4+ T cell count or pre-ART VL in VL rebound could be found in patients treated during acute HIV-1 infection who received a therapeutic HIV vaccine regimen comprising two doses of Ad26.Mos.HIV and two doses of MVA-Mosaic given over 48 weeks [36].

In addition, it seems that type of HLA of the host could influence VL rebound. Rosas-Umbert et al. [37] found that the number of HLA-associated polymorphisms in Gag predicted peak of viremia after ATI (*p* = 0.012) in patients recruited in a phase I therapeutic vaccine trial of a polyvalent MVA-B vaccine candidate. However, no influence of the presence of protective HLA class I alleles (B*57, B*27 or B*51) or number of HLA footprints in Gag were associated with time to rebound. These data differ from some of the findings of other groups that suggest that vaccinated participants with neutral HLA alleles had lower median VL 16 weeks after ATI than vaccinated participants did with protective HLA alleles or placebo participants with neutral HLA alleles. In this study, factors independently associated with lower VL 16 weeks after ATI included greater Gag sequence divergence from the vaccine sequence and decreased proportion of HLA-associated polymorphisms in Gag [35].

### 6.2. Immunological Responses Influence Viral Dynamics during ATI

#### 6.2.1. HIV-Specific CD4+ and CD8+ T Cell Responses

Lymphocyte CD4+ T cell proliferation after exposure to HIV antigens and IFN gamma secretion T lymphocytes are commonly used tests to measure immunogenicity in therapeutic HIV vaccine trials. Both parameters have been associated with VL control in chronic HIV infected patients [38,39], so the hypothesis that better T cell responses achieved after boosting the immune system could potentially control viral replication without ART, has been proven in different studies (Table 3). 

One of the first studies that described T cell responses in HIV vaccination used ALVAC vCP1452 in eleven chronic HIV infected patients. Absence of correlates between VL dynamics and T cell responses were described, but they were determined after ATI and not as predictors [40].

Therapeutic vaccination with autologous monocyte derived-dendritic cells (MD-DCs) loaded with autologous heat-inactivated HIV-1 in patients with non-advanced chronic HIV-1 infection showed a decrease in the magnitude and the breadth of the CD8+ T cell responses after the immunization period. However, there was no difference in the CD8+ T cell dynamics between virologic responders (decrease of >0.5 log in relation to baseline VL after immunization and stopping treatment) and non-responders. In contrast, responders had a weak but statistically significant increase in HIV-1 specific CD4+ lymphoproliferative responses (LPR) during immunization [41]. Eighty-one subjects were included in a four-arm strategy clinical trial with ALVAC vCP1452 (or placebo) with or without interleukin-2 (IL-2) infusions. There were no differences between groups in T cells responses. However, similar to the previous study, in all study patients, an inverse correlation between lymphoproliferative responses before stopping therapy and VL endpoint (VL < 5000 copies after 12 weeks of therapy withdrawal) was observed [42]. Lévy et al. described that vaccination with ALVAC vCP1433 and HIV-LIPO-6T + IL-2 markedly increased the percentage of patients who responded to HIV antigens as compared with non-vaccinated patients and in previous studies, where both HIV-1 specific CD4+ LPR and breadth of HIV-specific CD8+ T cell immune responses resulted in a positive correlation with time off treatment after ATI [43]. Similarly, vaccination with a DC-based therapeutic vaccine showed that plasma viral load (pVL) after ATI was inversely correlated with lymphoproliferative responses against HIV p24 protein and with the magnitude and breadth of HIV-1-specific T cell immune responses reached after immunization and before treatment interruption [44]. In contrast, these findings could not be reproduced in other clinical trials that did not find any differences in immunological responses in vaccinated and non-vaccination patients, neither an association between any immunological parameter studied and cumulative time off treatment [45] nor longer time to viral rebound [46].

Some studies with HIV therapeutic vaccines have demonstrated to be immunogenic, but with no beneficial effect on viral replication [47,48] and some others have observed paradoxical results. Immunogenicity was not induced in a multicenter randomized placebo-controlled study with ALVAC-HIV (vCP1452) [33]. Moreover, worse outcome was reported in vaccinated patients after ATI that was attributed to enhanced CD4+ T cell responses in the absence of protective CD8+ T cell responses. Authors have hypothesized that this imbalance in favor of CD4+ responses could represent a facilitator of HIV replication [49].

#### 6.2.2. HIV Antibodies

Only two studies have studied the relation of vaccine induced antibodies and VL rebound during ATI after immunization and opposite results have been encountered (Table 3). The ACTG A5197, a randomized, placebo-controlled trial of a therapeutic rAd5 HIV-1 gag vaccine, assessed the relationship of the viral reservoir with HIV-specific immune status and viral rebound kinetics. Neutralizing antibodies (NAb) titers at week 38 pre-ATI were not associated with the post ATI VL set point [50]. Conversely, in a later study using a therapeutic HIV vaccine with four modified peptides corresponding to highly conserved cross-clade regions on the major capsid protein, week 24/week 1 anti-C5/gp41 antibody titer fold change, pre ATI, was associated with a decrease of 1.35 log10 copies/mL in the vaccinated arm compared to placebo recipients at week 48 [51].

#### 6.2.3. Soluble Markers of Inflammation

Higher soluble markers of inflammation have been related to disease progression in HIV. Data addressing whether soluble inflammation markers could influence viral control dynamics after ATI has been assessed in a peptide-based therapeutic HIV-1 p24 Gag vaccine candidate (Vacc4x clinical trial). Better viral control was observed in vaccinated patients with higher levels of TNF alpha and IL-6 between week 28 and week 1. In contrast, the same observations in IFN-γ and TNF alpha secretion in the placebo group were not associated with better control of VL after ATI, suggesting a possible qualitative difference in the responses induced by the vaccine [52]. In relation to C-reactive protein (CRP), lower levels of viral load after immunization were seen in patients with lower C-reactive protein (CRP) week 28/week 1-fold change [51].

### 6.3. Viral Reservoir Size Influence Viral Dynamics during ATI

One of the major barriers to HIV functional cure is latency [1], and depleting this reservoir is one of the main goals of curative strategies. Siliciano et al. described the capacity of HIV-1 to latently infect CD4+ T cells and the persistence of an integrated viral DNA that could replicate despite suppressed plasma viremia [53,54]. This cell-based reservoir is responsible for viral rebound after antiretroviral treatment interruption [55] and it has been demonstrated that the HIV reservoir size can predict time to VL rebound after ATI [56,57]. Over the last years, the mechanisms of viral latency have been intensively studied, but many features still remain unknown. Another uncertainty is that measuring methods are not well standardized even though several different tests have been developed. The quantitative viral outgrowth assay (QVOA), which measures infectious units per million (UIPM) in resting CD4+ T cells is considered the gold standard for measuring latent HIV reservoir [58,59]; other measurements obtained by PCR have been proposed as surrogate biomarkers such as total DNA, also called cell-associated DNA (CA-DNA) [60], proviral, or integrated DNA [61,62] and cell-associated RNA (CA-RNA) [63].

#### 6.3.1. Quantitative Viral Outgrowth Assay (QVOA)

QVOA is the “gold standard” test for measuring the size of the latent reservoir. This assay requires extracting resting CD4+ T cells to activate them in order to induce HIV replication by any latently infected cell. Although it has positive features, it has several important disadvantages such as time consuming, costs, and the need for many viable cells [58,59] therefore, it is not widely used in clinical trials. Angel and Cols [46] conducted a randomized double-blind placebo controlled study evaluating a canarypox virus-based vaccine in combination with a gp120-depleted HIV-based vaccine and found a delay in time to rebound (TtR) and time to restart ART in the participants receiving the study product. Afterward, investigators conducted a reservoir substudy [64] to evaluate the effect of the vaccines on the viral reservoir and found no effect in the size of the reservoir between baseline and after last vaccination and no correlations between the size of the viral reservoir with TtR or time to restart ART. Another one-arm study evaluating the effect on the latent reservoir of a peptide-based vaccine combined with a human granulocyte macrophage stimulating factor and a latency reverse (romidepsin) agent found a 38% decrease of UIPM from screening to six weeks after the romidepsin, but did not prolong TtR [65].

#### 6.3.2. Total DNA

Total DNA is the quantification of all forms of HIV-DNA, integrated and unintegrated, coding for both competent and defective viruses reflecting the global size of the reservoir giving an overview of its distribution in the body at all stages of the disease [66]. Several studies testing different therapeutic based-vaccine strategies (alone, boosted or combined) [33,37,44,45,50,65,67] (Table 4), have measured total DNA as a reservoir biomarker and found different results when assessing the effect on pVL during ATI. Some of these studies, one conducted in patients that started ART during acute infection [45], have described a correlation between a smaller size of baseline CA-DNA and longer time to resume ART after ATI [33,50], but some others found no relation [37,44,67]. Neither of these studies found any significant effect on CA-DNA of any of the studied products, although a study evaluating a DC-based vaccine found that the magnitude of T cell responses measured by IFNγ ELISPOT during the vaccination period was an independent predictor of total DNA during ATI and also found a correlation between total DNA levels and pVL set point during ATI [44]. Leth and Cols conducted a single arm study with the overall objective to assess the effect on the size of the reservoir of combining a peptide-based vaccine with a macrophage stimulating factor as adjuvant and romidepsin as a latency reverse agent and found a significant reduction between baseline and after intervention total DNA, but did not prolong TtR [65].

#### 6.3.3. Proviral DNA

During the viral replication, only a fraction of total DNA is integrated, and an even smaller fraction is capable of producing infectious virus [53]. Memory CD4+ T cells contain more integrated HIV DNA than naive cells, suggesting that these cells are the largest proportion of the reservoir [68] and could be responsible for maintenance and replenishment. It has been described that ART untreated individuals have different levels of total DNA than integrated [69], with an increase in the ratio between both during viral replication [70] denoting that these measurements are not interchangeable. Only a few therapeutic vaccine studies including ATI have analyzed integrated DNA as a reservoir biomarker [37,44,48,71] (Table 4).

A clinical trial using a poxvirus-based vaccine (MVA-B) alone or in combination with a drug to reactivate latent HIV-1 (disulfiram) assessed the impact on latent reservoir and pVL rebound during ATI and found that only participants receiving vaccinations alone had a marginal delay in TtR after treatment interruption, but failed to have an impact on the viral reservoir [48]. When analyzing the potential predictors of vaccine outcomes, these investigators found that proviral DNA was associated with a peak of pVL during rebound and that after 12 weeks of ATI, proviral DNA increased a median of 3.1 fold in all of the tested subjects. These proviral DNA levels at week 12 after ATI strongly correlated with levels of proviral DNA before vaccinations, but there were no differences between placebo and vaccine recipients. When assessing if the referred markers had any impact on pVL kinetics, the only variable that was independently associated with TtR was proviral DNA before any vaccination [37]. Similarly, another clinical trial evaluating a therapeutic vaccine in patients that started ART during acute infection using DNA priming (GOVX-B11) and MVA boosting showed no changes in proviral-DNA after interventions, but found that TtR tended to be shorter in participants with the highest levels of this proviral DNA [71].

An autologous DC-based therapeutic vaccine (DCV2) was able to decrease the peak viral set point after ATI [72] and in a substudy, Andrés and Cols investigated the changes in the latent reservoir before and after vaccination and its correlation with different biomarkers during treatment interruption [44]. At post-vaccination phase after 12 weeks on ATI, there was a significant increase of integrated HIV DNA in the control group that was not observed in the study group. They also found some correlations between the reservoir and T cell responses and lymphoproliferation concluding that the T cell immune responses elicited by DCV2 were associated with the reservoir size after vaccinations and delayed the increase of integrated DNA during ATI [44].

#### 6.3.4. Cell-Associated RNA

More than 40 viral RNA are produced in HIV infected cells [63]. CA-RNA measures HIV transcription in infected cells or the viral entry and indicates the degree of ongoing viral replication [73]. It is a reservoir biomarker that has shown to be significantly more sensitive for predicting virological failure than plasma viremia and has been suggested as a predictor of the efficacy of latency reversal [74].

Only very few therapeutic vaccine clinical trials have assessed CA-RNA as a reservoir marker and have studied if there are any relations with pVL dynamics during ATI [48,50] (Table 4). A double-blind randomized placebo controlled study evaluating an adenovirus type 5-based vaccine found a lower viral rebound set-point in the vaccine arm [75] and, in a substudy where they explored the impact of the vaccines in the reservoir, they found that vaccination did not change the reservoir, but levels of CA-RNA correlated with total DNA and residual viremia, and a correlation between having unfavorable HLA alleles and higher levels of CA-RNA. A higher viral set-point during ATI was positively correlated with CA-RNA pre-ATI [50].

### 6.4. OMICS Data—A New Approach

Systems biology is a scientific approach to study complex biological systems as a whole, which has gained great popularity in our century due to the almost exponential increase in computational capacity in the last decades. The integrative study of a large set of biological data is referred to by the suffix—omics—such as genomics (genes), proteomics, (proteins), metabolomics (metabolites), microbiomics (microbiome) or transcriptomics (RNA), which is a fundamental part of systems biology. The application of such methodology in the development of vaccines gave birth to systems vaccinology, a young discipline that seems to be a useful tool in a number of stages of modern vaccine development strategies [76] including the prediction of vaccine response [77,78,79,80,81,82,83].

Although the intersection of systems vaccinology and therapeutic HIV-1 vaccine research is very small thus far, there are some interesting data available. Interestingly, all available data comes from studies performed with DC-based vaccines.

The pioneer study in this area was published five years ago by de Goede et al. [84] where the authors demonstrated that a therapeutic vaccine was able to elicit significant changes in transcriptomic patterns in PBMC, in great part due to the u-regulation of genes related to—both innate and adaptive—immune activation. However, due to the lack of response to the vaccine in question [85], the authors could not relate omics data to virological outcome.

A recent substudy of the DALIA trial [86], is the only publication yet where post-vaccination changes of transcriptomic patterns in whole blood were correlated to HIV rebound dynamics during ATI [87]. The virological end-point assessed in this study was the peak VL observed during the 24-week-long ATI, and the authors investigated if changes in whole blood gene expression at a time point four weeks after vaccination and eight weeks before ATI may correlate to this parameter. They observed that the expression of 45 genes (of which 26 were related to inflammation and 13 to neutrophil lymphocytes) were directly correlated to post-ATI peak VL, while there was an inverse correlation in the case of 12 genes (seven of which related to T cell activation). These data suggest a better viral control in patients who mainly mobilized genes related to specific immune response to the vaccine, as compared to the ones who responded with the predominant expression of inflammatory genes.

Our group analyzed the relationship between post-vaccination transcriptomic patterns and virological response [88] in participants of the DCV2 trial [72]. In this trial, responders were defined on the basis of a greater than 1 log10 copies/mL drop in VL set point (measured at week 12 of ATI) with respect to the pre-ART values. We could not identify any differentially expressed genes between responders and non-responders. However, by gene set enrichment analysis, we identified several significantly deregulated gene sets related to immune processes between these two groups. Among the gene sets that were upregulated in responders, we found genes related to both innate and specific immune response, but some of these (such as TNF-alpha signaling via NFkB, IL-6-JAK-STAT3 signaling, IL2-STAT5 signaling) play important roles in the differentiation and/or activation of T- lymphocytes. At the same time, among the downregulated gene sets—along with a series of cell-cycle regulating genes—we found a response to interferon-alpha, an important player in innate antiviral immunity.

Both of these studies underline the importance of the predominance of specific immune response in order to achieve better response to vaccination, similar to what was observed by others in the case of different preventive vaccines [77,79] including one for HIV-1 [82]. Although more data are needed before these observations can be of practical use, considering the growing popularity and fast evolution of systems vaccinology [89], omics studies will surely form an important part of therapeutic vaccine research in forthcoming years.

## 7. Conclusions

Many HIV therapeutic vaccines have been evaluated and some others are currently in clinical trials. In all of these studies, ATI is the main intervention to assess efficacy. Different surrogate markers of viral control after ATI have been studied with different outcomes. There have been classical assessments such as host factors, immunologic assays, and reservoir measurements and more recent approaches such as omics data, but still not one seemed to robustly predict post-intervention viral control, hence ATI seems currently irreplaceable to evaluate the effect of therapeutic vaccines alone or in combination, even though there are potential related risks. Further research is necessary for a reliable surrogate biomarker focused on improving classical strategies and pursuing new approaches.

## Figures and Tables

**Table 1:** Summary of potential adverse effects analyzed and reported in ATI studies.

Clinical Risks	
Acute Retroviral Syndrome	Ruiz [17] Fagard [16] Tarwater [18] Ortiz [19]
Thrombocytopenia	Tarwater [18] Ananworanich [20] Piketty [21]
Cardiovascular, renal, or hepatic disease	SMART study group [6]
Neurological events	Price [23]
Opportunistic disease	SMART study group [6]
Death	SMART study group [6]
Increase in inflammation markers	Kuller [22]
Antiretroviral Drug Resistance	
Antiretroviral drug resistance	Arnedo [30] Lau [10]
Effects in Virologic and Immunologic Parameters	
Integrated HIV DNA (viral reservoir)	Clarridge [25] Salantes [26] Strongin [27] Papasavvas [28] Montserrat [29]
HIV Transmission	
HIV transmission reported cases	Lelièvre [31] Ugarte [32]

Note: ATI: Analytical antiretroviral treatment interruption.

**Table 2:** Summary of influences of host, viral load, and CD4+ T cell counts on viral load rebound.

CD4+ T Cell Count and PreART Viral Load	
Chronic HIV-1 Infected Patients	
A lower nadir CD4+ T cell count and higher preART viral load had a shorter time to ART resumption	Autran [33]
CD4+ T cell count and higher preART viral load correlated with time to ART resumption	Huang [34]
preART viral load correlated with shorter time to viral load rebound	Li [35]
Patients Treated During Acute HIV-1 Infection	
No evidence of effect of CD4+ T cell count or preART VL	Colby [36]
HLA	
The number of HLA-associated polymorphisms in Gag predicted peak of viremia after ATI. No influence of the presence of protective HLA class I alleles (B*57, B*27 or B*51) or number of HLA footprints in Gag were associated with time to rebound of viral load	Rosas-Umbert [37]
Participants with neutral HLA alleles had lower median VL 16 weeks after ATI than did vaccinated participants with protective HLA alleles or placebo participants with neutral HLA alleles. Factors independently associated with lower VL 16 weeks after ATI included greater Gag sequence divergence from the vaccine sequence and decreased proportion of HLA-associated polymorphisms in Gag	Li [35]

Note: ART: antiretroviral therapy; VL: viral load; ATI: analytical treatment interruption; HLA: human leukocyte antigen.

**Table 3:** Immunological responses and inflammation.

Patients Treated in Chronic HIV-1 Infection	
Cell mediated immune responses	
Slightly higher CD4+ LPR in patients with lower VL No relation in magnitude and breadth of CD8+ T cell responses with VL	Garcia [4]
Inverse correlation between CD4+ LPR and VL	Kilby [5]
Positive correlation of both, CD4+ LPR and CD8+ breadth responses, with time off ART	Lévy [6]
Inverse correlation between CD4+ LPR and CD8+ magnitude and breadth and VL	Andrés [7]
No association between CD4+ and CD8+ responses and longer TtR	Angel [9]
No association of CD4+ LPR with time off ART	Pialoux [10]
No association of T cell responses and VL	Mothe B [11]
High CD4+ responses but absence of CD8+ responses associated to higher VL and shorter time to restart ART	Papagno [13]
Patients Treated During Acute HIV-1 Infection	
No association between CD4+ and CD8+ responses and time off ART	Goujard [8]
HIV antibodies	
No association of NAb (pre-ATI) with VL set point	Li [14]
anti-C5/gp41 increase from week 1-preATI associated with low VL	Huang [15]
Inflammation markers	
VL control associated with increase in TNFα and IL-6 from week 1-preATI	Huang [16]
Higher VL associated with increase in CRP from week 1-preATI	Huang [15]

Note: LPR: lymphoproliferative responses; VL: viral load; ART: antiretroviral therapy; NAb: neutralizing antibodies; ATI: analytical treatment interruption; TNFα: tumor necrosis factor alpha; CRP: c-reactive protein.

**Table 4:** Evidence summary of latent HIV-1 reservoir effect on viral load dynamics.

Latent Viral Reservoir Measurements	
Patients Treated When Chronically HIV-1 Infected	
QVOA	
No evidence of effect on UIPM	Angel [14]
38% decrease of UIPM after interventions but did not prolong TtR	Leth [15]
Total (cell-associated) DNA	
Lower baseline total DNA content was associated with longer time to restart ART	Autran [17]
Higher baseline level of total DNA correlate with higher pVL set point during ATI	Li [18]
Reduction of total DNA after interventions but did not prolong TtR	Leth [15]
Total DNA levels increased during ATI in both study and control group without significant differences Correlation between total DNA levels during ATI and the magnitude of T cell responses during vaccination Correlation between total DNA levels and pVL set point during ATI	Andrés [19]
No evidence of effect on total DNA	Rosas-Umbert [21]
No evidence of effect on total DNA—One participant showed a 2-fold reduction during ATI (the only participant carrying HLA alleles associated with natural HIV control)	Mothe [22]
Proviral (Integrated) DNA	
Baseline proviral DNA was associated with peak of pVL during rebound Baseline proviral DNA was independently associated to TtR	Rosas-Umbert [21]
No evidence of effect on proviral DNA after disulfiram but substudy (Rosas-Umbert)	Mothe [26]
Higher baseline proviral DNA levels trended to correlate with shorter TtR	Thompson [27]
Correlation between integrated DNA levels during ATI and the magnitude of T cell responses during vaccination Correlation between integrated DNA levels and pVL set point during ATI	Andrés [19]
Cell-associated RNA (CA-RNA)	
Higher baseline level of CA-RNA correlate with higher pVL set point during ATI	Li [18]
No evidence of effect on CA-RNA after disulfiram	Mothe [26]
Patients Treated During Acute HIV-1 Infection	
Total (cell-associated) DNA	
Lower total DNA baseline level was associated with a longer time off ART Low total DNA level at ART interruption was associated with a low peak of pVL	Goujard [20]
Patients Treated When Chronically HIV-1 Infected	
QVOA	
No evidence of effect on UIPM	Angel [14]
38% decrease of UIPM after interventions but did not prolong TtR	Leth [15]
Total (cell-associated) DNA	
Lower baseline total DNA content was associated with longer time to restart ART	Autran [17]
Higher baseline level of total DNA correlate with higher pVL set point during ATI	Li [18]
Reduction of total DNA after interventions but did not prolong TtR	Leth [15]
Total DNA levels increased during ATI in both study and control group without significant differences Correlation between total DNA levels during ATI and the magnitude of T cell responses during vaccination Correlation between total DNA levels and pVL set point during ATI	Andrés [19]
No evidence of effect on total DNA	Rosas-Umbert [21]
No evidence of effect on total DNA—One participant showed a 2-fold reduction during ATI (the only participant carrying HLA alleles associated with natural HIV control)	Mothe [22]
Proviral (Integrated) DNA	
Baseline proviral DNA was associated with peak of pVL during rebound Baseline proviral DNA was independently associated to TtR	Rosas-Umbert [21]
No evidence of effect on proviral DNA after disulfiram but substudy (Rosas-Umbert)	Mothe [26]
Higher baseline proviral DNA levels trended to correlate with shorter TtR	Thompson [27]
Correlation between integrated DNA levels during ATI and the magnitude of T cell responses during vaccination Correlation between integrated DNA levels and pVL set point during ATI	Andrés [19]
Cell-associated RNA (CA-RNA)	
Higher baseline level of CA-RNA correlate with higher pVL set point during ATI	Li [18]
No evidence of effect on CA-RNA after disulfiram	Mothe [26]
Patients Treated During Acute HIV-1 Infection	
Total (cell-associated) DNA	
Lower total DNA baseline level was associated with a longer time off ART Low total DNA level at ART interruption was associated with a low peak of pVL	Goujard [20]

QVOA: quantitative viral outgrowth assay; UIPM: Unit infectious per million; TtR: time to rebound; ART: antiretroviral therapy; pVL: plasma viral load; ATI: analytical treatment interruption; HLA: human leukocyte antigen.
